# Density homogeneity as a crucial CT indicator for differentiating malignant and benign subcentimeter solid pulmonary nodules: A retrospective multi-center study

**DOI:** 10.1186/s13244-026-02301-9

**Published:** 2026-05-16

**Authors:** Wen-tao Zhang, Hui Gan, Wei Luo, Min Zhao, Can Ding, Xue-feng Jiang, Ting Fu, Fa-jin Lv, Zhi-gang Chu

**Affiliations:** 1 Department of Radiology, The First Affiliated Hospital of Chongqing Medical University, Chongqing, China; 2 Department of Radiology, The Second Affiliated Hospital of Army Medical University, Chongqing, China; 3 Department of Radiology, The Chongqing University Three Gorges Hospital, Chongqing, China

**Keywords:** Lung neoplasms, Tomography (X-ray computed), Diagnosis (differential)

## Abstract

**Objectives:**

To determine the significance of density homogeneity in differentiating malignant and benign subcentimeter solid nodules (SNs).

**Materials and methods:**

Between January 2018 and July 2024, 735 subcentimeter malignant SNs (SMSNs) and 814 subcentimeter benign SNs (SBSNs) from Center 1 (training set), as well as 244 SBSNs and 163 SMSNs from two other centers (validation set), were included. Patients’ clinical characteristics (e.g., age, gender) and CT features of lesions (e.g., density homogeneity, diameter) were analyzed and compared, focusing on assessing the significance of density homogeneity in differential diagnosis. The optimal cutoff value of standard deviation (SD) of mean CT value for distinguishing visually heterogeneous and homogeneous SNs was calculated and validated, respectively.

**Results:**

The optimal SD cutoff for density heterogeneity was 57.3 HU, with an area under the curve (AUC) of 0.928 (*p* < 0.001) in the validation set. In training set, heterogeneous density demonstrated the highest predictive efficiency among all CT features (AUC: 0.722), and significantly improved the performance of predictive model after incorporating this indicator (AUC from 0.698 to 0.824, *p* < 0.001). The model indicated that heterogeneous density, lobulation, spiculation, and air bronchogram sign were independent predictors of SMSNs (all *p* < 0.05). In validation set, density heterogeneity also demonstrated the highest predictive efficiency (AUC: 0.738), and significantly enhanced the predictive model’s performance (AUC from 0.679 to 0.831, *p* < 0.001).

**Conclusions:**

Heterogeneous subcentimeter SNs, especially those with lobulation, spiculation, or air bronchogram sign, should raise a high suspicion of malignancy; therefore, close monitoring is required.

**Critical relevance statement:**

Accurately differentiating malignant and benign solid pulmonary nodules at the subcentimeter stage is still challenging; the present study confirmed that heterogeneous density of lesions is a stable and reliable radiological predictor of malignancy.

**Key Points:**

- Benign and malignant subcentimeter solid pulmonary nodules share considerable overlaps in CT morphological features, making differential diagnosis challenging.
- Heterogeneous lesions were significantly more common in SMSNs than in benign ones, particularly in the group of nodules ≤ 8 mm.
- Heterogeneous density exhibited superior efficiency than lobulation, spiculation, pleural indentation, vacuole sign and other morphological features in differentiating subcentimeter SNs.
- Heterogeneous subcentimeter SNs, particularly those with lobulation, spiculation, or air bronchogram sign, should be highly suspected for malignancy.

**Graphical Abstract:**

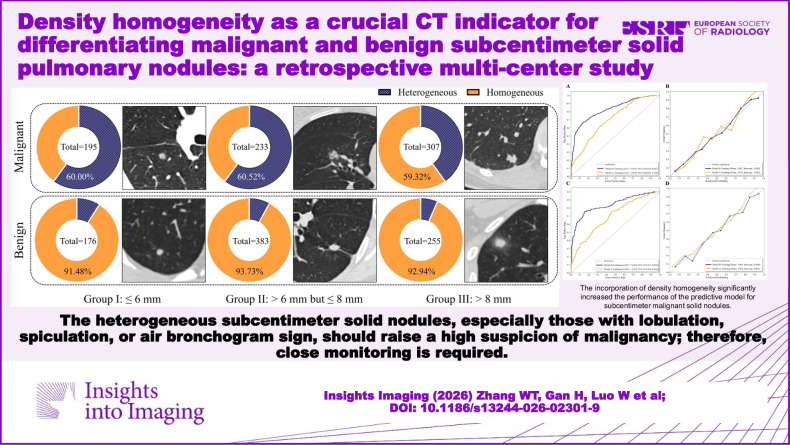

## Introduction

Lung cancer remains the leading cause of cancer-related mortality worldwide [1]. It has been confirmed that tumors presenting as solid nodules (SNs) are associated with a poorer prognosis compared with those manifesting as ground-glass nodules on computed tomography (CT) images [2, 3]. This emphasizes the clinical importance of the early and accurate identification of malignant SNs. Numerous studies have reported that patients with non-small cell lung cancer (NSCLC) at T1a stage (≤ 1 cm) manifested as SNs have significantly higher 5-year overall survival rates and lower incidences of lymph node metastasis compared with those with lesions greater than 1 cm [4–7]. Therefore, accurately distinguishing subcentimeter malignant SNs (SMSNs) from subcentimeter benign SNs (SBSNs) is critical for improving patient prognosis.

However, the majority of detected subcentimeter SNs are benign and share substantial overlap in CT features with the malignant ones [8, 9]. Additionally, the traditional CT features used for diagnosing lung cancers are less commonly observed in SMSNs [10], which further complicates their differentiation. Although a few studies have successfully developed predictive models for SMSNs based on CT features, there is significant variability in the efficiencies of these models (areas under the curve [AUC] range: 0.747–0.875) and the CT features [11, 12]. Moreover, some CT parameters were controversial in different studies. These discrepancies may be attributed to insufficient sample sizes and subjective bias in CT feature evaluation. While some studies have established radiomic prediction models recently, the poor reproducibility and clinical applicability of radiomics remain challenging [13, 14]. Therefore, further exploration and validation with larger cohorts are required to identify more applicable, stable, and reliable CT indicators.

During the progression of lung cancer, tumor cells or secreted mucus gradually fill the alveolar spaces. Simultaneously, fibrous tissue proliferation increases, leading to the collapse of the alveolar structure [15, 16]. As a result, CT attenuation of the lesion increases and eventually reaches the threshold for a solid lesion [17–19]. Therefore, when tumors are still in an intermediate stage of this gradual progression, and the alveolar spaces have not been fully replaced, their density may be heterogeneous. In contrast, most benign SNs, such as fibrotic nodules, granulomas and benign tumors, lack airspaces or contain only a small amount, generally presenting as homogeneous hyperdensity on lung window CT images [20, 21]. Thus, the differences in density characteristics of malignant and benign SNs, correlated with their diverse histopathological features, may provide new perspectives for differential diagnosis. However, this potential density indicator for differentiating subcentimeter SNs has not yet been thoroughly validated.

In clinical practice, it was found that lesions with heterogeneous density were more commonly observed in SMSNs. Based on this observation, we hypothesize that density homogeneity may serve as a key indicator for differentiating subcentimeter SNs. Therefore, this study aims to investigate the significance of density homogeneity in distinguishing SMSNs from SBSNs, with the goal of improving the accurate early detection of potentially high-risk nodules.

## Materials and methods

This multi-center retrospective study received approval from the ethics committees of the First Affiliated Hospital of Chongqing Medical University (2025-117-01), the Second Affiliated Hospital of Army Medical University (2022-196-01), and the Chongqing University Three Gorges Hospital (2023 NO.104). Informed consent was waived due to the retrospective nature of this study.

### Patients

This study retrospectively reviewed patients with pathologically confirmed pulmonary lesions and those with stable nodules on follow-up chest CT scans from the First Affiliated Hospital of Chongqing Medical University (Center 1; *n* = 19,756), the Second Affiliated Hospital of Army Medical University (Center 2; *n* = 2528), and the Chongqing University Three Gorges Hospital (Center 3; *n* = 1558) between January 2018 and July 2024. Their chest CT images were manually searched and reviewed in the Picture Archiving and Communication System (PACS) workstation.

The patients enrolled in this study required the following conditions to be satisfied: (1) availability of chest CT scans performed within two weeks prior to surgical resection specifically for lesions confirmed by pathological examinations; (2) a CT follow-up interval of ≥ 2 years specifically for lesions confirmed by follow-up; (3) lesions identified as SNs; and (4) lesions had a diameter ≤ 1 cm. A SN was defined as a nodule with pure soft-tissue attenuation that completely obscures the underlying bronchial and vascular structures [22]. Regarding the SN with minimal peripheral ground-glass opacity (GGO) surrounding the lesion, it was classified as a SN with halo sign rather than being categorized as a part-solid nodule according to Fleischner Society’s recommendations [22]. The evaluation of SN was independently performed by a fellowship-trained thoracic radiologist (W.T.Z., with 6 years of experience in chest imaging; Radiologist A) and a senior thoracic radiologist (H.G., with 15 years of experience in chest imaging; Radiologist B) under lung window settings (level: −600 HU; width: 1200 HU), and any discrepancies between the radiologists were resolved through consensus. The exclusion criteria were as follows: (1) patients without complete clinical data; (2) slice thickness of CT images greater than 1.0 mm; and (3) significant artifact or noise on CT images affecting evaluation.

**Fig. 1:**
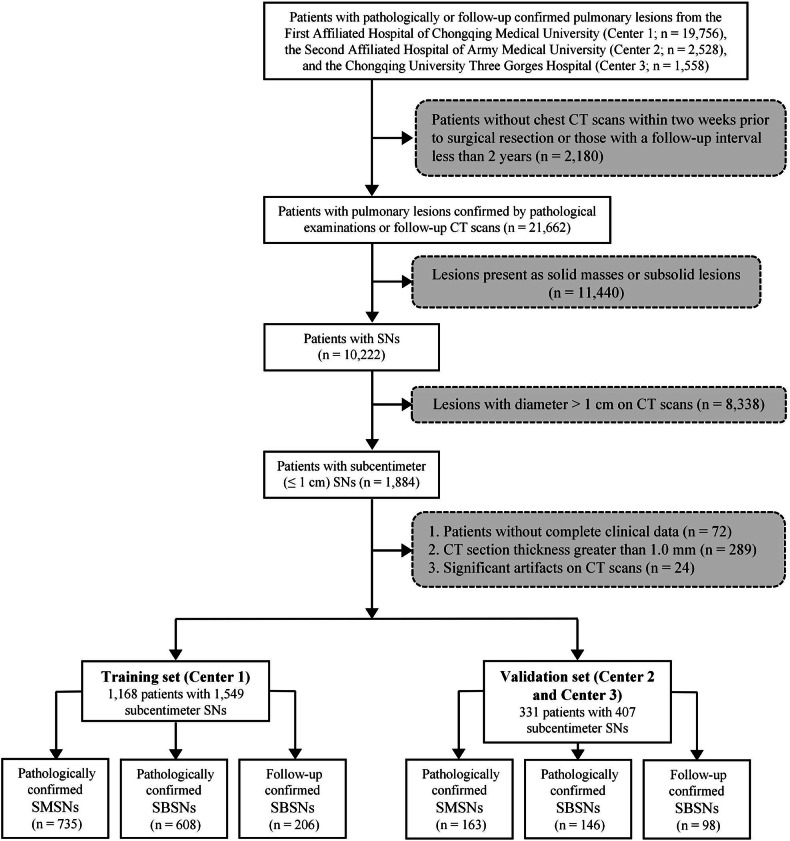
The flowchart of patient selection. CT, computed tomography; SNs, solid nodules

Finally, 512 patients with 735 SMSNs and 656 patients with 814 SBSNs from Center 1 were included as the training set, whereas 190 patients with 244 SBSNs and 141 patients with 163 SMSNs from Center 2 and Center 3 were included as the validation set (Fig. 1).

### CT examinations

The chest CT scans were performed using the following CT scanners: SOMATOM Perspective (Siemens Healthineers), Discovery CT750 HD (GE Healthcare), SOMATOM Definition Flash (Siemens Healthineers), SOMATOM Force (Siemens Healthineers), OPTIMA CT660 (GE Healthcare), and Aquilion ONE pureViSION (Canon Medical System). All patients were placed in a supine position with raised upper limbs and were asked to hold their breath after deep inspiration for better exposure. The scan range was from the thoracic entrance to the costophrenic angle. Scanning parameters included a tube voltage of 110–120 kVp, a reference tube current of 50–140 mAs (automatic tube current modulation technology), both reconstruction slice thickness and interval of 0.625 or 1.0 mm (the slice thickness and interval were identical in the same scanner), a 512 × 512 matrix, a pitch of 0.975–1.2, a field of view of 350–400 mm, and a rotation time of 0.5 s, using medium-sharpness reconstruction algorithm (Table S1).

### Clinical data and image analysis

The electronic health records system was searched to collect the patients’ age, gender, smoking history, family history of lung cancers, history of diabetes, history of hypertension, history of malignant tumors, and tumor markers (including carcinoembryonic antigen [CEA], cytokeratin 19 antigen [CYFRA 21-1], neuronal-specific enolase [NSE], gastrin-releasing peptide precursor [pro-GRP], squamous cell carcinoma antigen [SCC-Ag]).

CT features of all lesions were evaluated, including: (a) diameter (calculated as the mean of the longest diameter and the perpendicular diameter on axial CT images), (b) location (upper lobe, middle or lower lobe), (c) shape (round, oval, or irregular), (d) density homogeneity (heterogeneous or homogeneous), (e) boundary (well-defined or ill-defined), (f) margin (smooth or coarse), and (g) other manifestations (lobulation, spiculation, vacuole sign, pleural indentation, air bronchogram sign, and halo sign). All measurements, except density homogeneity, followed the Fleischner Society’s recommendations for measuring CT features of pulmonary nodules [22].

**Fig. 2:**
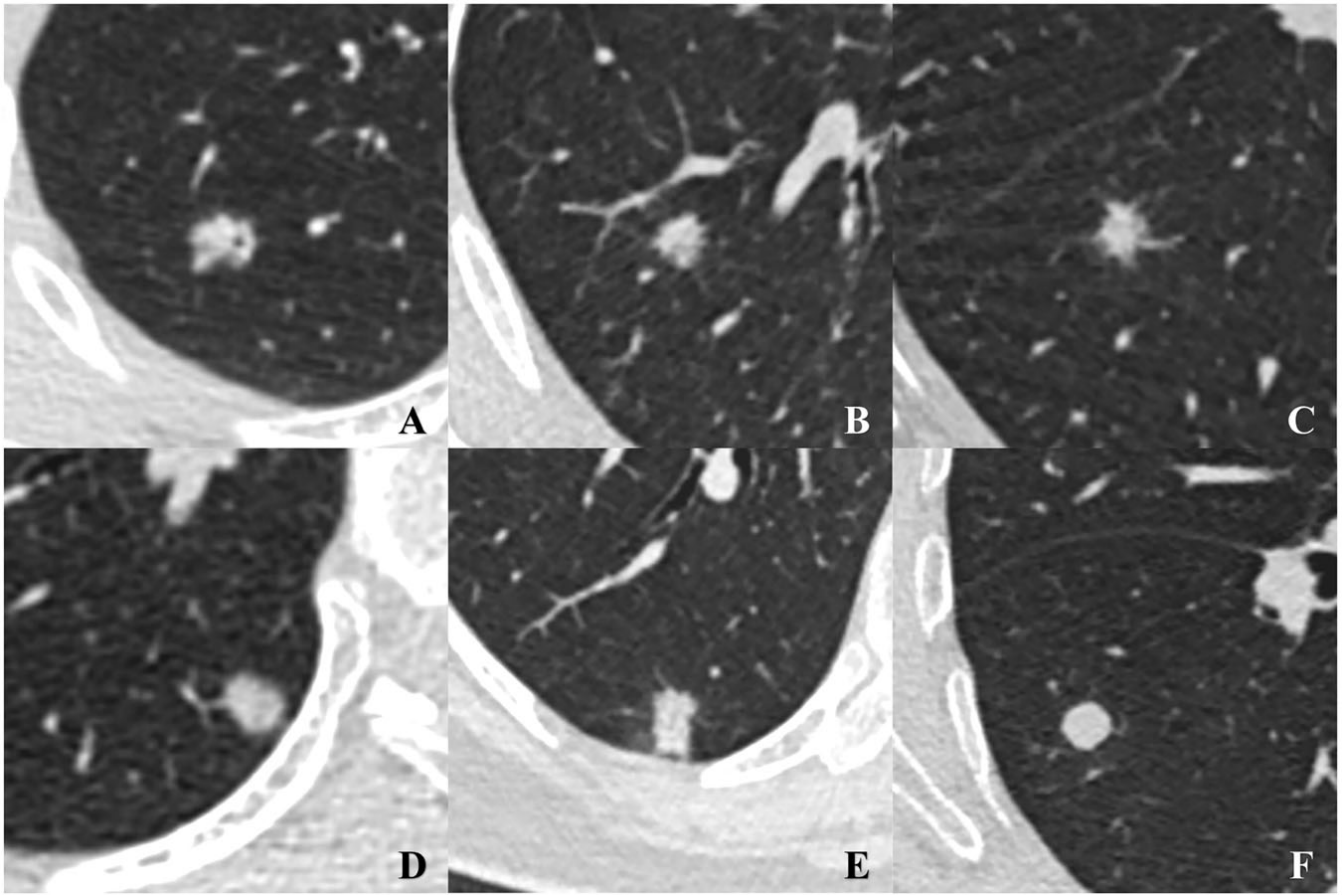
SNs with different density homogeneity. **A**–**E** Heterogeneous SNs: these nodules overall exhibit nonuniform soft tissue density on axial CT images, with interspersed regions of relatively high and low attenuation. **F** Homogeneous SN: this nodule exhibits pure soft tissue attenuation on axial CT image, without any region of relatively low attenuation. SN, solid nodule; CT, computed tomography

Based on density homogeneity, SNs were categorized into heterogeneous and homogeneous subtypes. A heterogeneous SN was defined as a nodule demonstrating overall nonuniform soft-tissue attenuation on thin-section CT (TSCT) image, with interspersed regions of relatively higher and lower attenuation. The internal vacuole and bronchus as lower attenuation regions were not regarded as evidence for determining heterogeneous SNs, as they are incidental and do not reflect the histological heterogeneity of lesions. By contrast, a homogeneous SN showed uniform density, presenting as a lesion with pure soft-tissue attenuation (Fig. 2). After visual assessment, the mean CT value and corresponding standard deviation (SD) of each lesion were measured on the most representative axial image: the slice with the most prominent heterogeneity for heterogeneous SNs, or the largest slice for homogeneous SNs. All CT value measurements were performed using a region of interest (ROI). The ROI was delineated to cover approximately 70% of the nodule’s area on its most representative axial slice (Fig. S1). If a vacuole or air bronchogram sign was present within the nodule, an irregular ROI was manually delineated while carefully avoiding these air-containing structures.

The previously mentioned radiologists (Radiologist A and Radiologist B) who were blinded to the final pathological results and clinical data, independently evaluated the TSCT images using the lung window setting via PACS workstation. Additionally, because density homogeneity is a novel feature, a third radiologist (Z.G.C., with 18 years of experience in chest imaging; Radiologist C) also assessed this parameter, and all three radiologists re-evaluated it one week later for calculating the intra-observer agreement. Any discrepancies in categorical variables between the three radiologists were resolved by consensus, which involved a joint review to reach an agreement, based on multi-planar reconstruction images. The values for continuous variables were calculated as the mean of the measurements from both Radiologist A and Radiologist B.

### Statistical analysis

All statistical analyses were conducted using SPSS (version 25.0, IBM) and Python (version 3.8.19). The intraclass correlation coefficient (ICC) and Fleiss’ kappa were used for continuous and categorical variables, respectively. Continuous variables were expressed as the mean ± SD or the median (range), while categorical variables were expressed as a number and percentage. Differences between SBSNs and SMSNs in continuous variables were assessed using the Mann-Whitney U test, whereas differences in categorical variables were compared using Pearson’s chi-square test.

The optimal cutoff threshold of the SD of mean CT value for discriminating heterogeneous from homogeneous SNs was determined by Receiver operating characteristic (ROC) analysis and maximizing the Youden index. The predictive value of clinical characteristics and CT features was evaluated using ROC analysis, and the AUC was used to evaluate the predictive performance. To identify independent predictors and avoid overfitting, variables with a *p* < 0.05 in the univariate analysis were further selected by the least absolute shrinkage selection operator (LASSO) regression analysis to confirm their independence. The retained variables (with non-zero coefficients in the LASSO regression analysis) were included in the multivariable analysis to identify the independent risk predictors and to construct predictive models. We used mixed-effects logistic regression with ‘patient ID’ as the random effect to address within-patient lesion correlation and performed bootstrap internal validation with 1000 repetitions for optimism-corrected AUCs. Brier score and calibration curves were constructed to evaluate the calibration performance of the model prediction. Model comparisons were based on the DeLong test and net reclassification index (NRI). A *p*-value less than 0.05 was considered statistically significant.

## Results

### Inter-observer agreement for CT features

For all lesions in both the training and validation sets, the inter-observer agreement for nodule diameter, mean CT value, and SD of mean CT value was excellent (ICC = 0.892–0.924). For the categorical indicators, the inter-observer agreement for assessing shape, margin, boundary, spiculation sign, pleural indentation, vacuole sign, air bronchogram sign, halo sign, and calcification was excellent (κ = 0.801–1.000), and that for lobulation was substantial (κ = 0.770 [95% CI: 0.734–0.807]). Regarding density homogeneity, inter-observer agreement (κ = 0.851 [95% CI: 0.827–0.873]) and intra-observer agreement (κ = 0.926–0.951) were almost perfect (Table S2). Radiologists A, B, and C had 60, 45, and 32 cases with discrepant classifications of density homogeneity between their twice assessments, respectively. The inter-observer discrepancies among three radiologists were found in 179 cases, which were then re-reviewed and adjudicated by consensus, resulting in a final classification of 109 heterogeneous and 70 homogeneous nodules.

### Patients’ clinical characteristics and CT features of lesions

The distribution of pathological diagnosis across the training and validation sets is summarized in Table 1. Within the benign nodules, 206 in the training set and 98 in the validation set were confirmed through radiological follow-up, and the other lesions were by pathological examinations. Tables 2 and S3 summarize the patients’ clinical characteristics in the training and validation sets, respectively. In the training set, diabetes was more common in patients with SBSNs compared to those with SMSNs (15.09% vs 4.10%, *p* < 0.001).

**Table 1:** Distribution of pathological diagnosis of lesions in the training and validation set

Category	Training set (*n* = 1549)	Validation set (*n* = 407)
SBSN	814 (100.00)	244 (100.00)
Fibrous-hyaline degeneration	264 (32.43)	76 (31.15)
Granulomas	257 (31.57)	48 (19.67)
Hamartomas	58 (7.13)	14 (5.74)
Lymph nodes	24 (2.95)	6 (2.46)
Sclerosing pneumocytomas	5 (0.61)	2 (0.82)
Lesions confirmed by follow-up	206 (25.31)	98 (40.16)
SMSN	735 (100.00)	163 (100.00)
Non-MACs	665 (90.48)	144 (88.35)
IACs	373 (50.75)	91 (55.83)
MIAs	158 (21.50)	28 (17.18)
AISs	134 (18.23)	25 (15.34)
MACs	43 (5.85)	18 (11.04)
SCCs	25 (3.40)	1 (0.61)
SCLCs	2 (0.27)	0 (0.00)

Note: Values are expressed as a number (%)

*SBSNs* subcentimeter benign solid nodules, *SMSNs* subcentimeter malignant solid nodules, *non-MACs* non-mucinous adenocarcinomas, *IACs* invasive adenocarcinomas, *MIAs* minimally invasive adenocarcinomas, *AISs* adenocarcinomas in situ, *MACs* mucinous adenocarcinomas, *SCCs* squamous cell carcinomas, *SCLCs* small cell lung cancers

**Table 2:** Comparison of clinical characteristics between patients with SBSNs and SMSNs in the training set

Characteristics	SBSNs	SMSNs	*p*-value	ROC analysis	
	(*n* = 656)	(*n* = 512)		AUC (95% CI)	*p*-value
Age (years)	56.31 ± 11.71	56.43 ± 12.08	0.911		
Gender			0.582		
Male	291 (44.36)	218 (42.58)			
Female	365 (55.64)	294 (57.42)			
Smoking history	173 (26.37)	113 (22.07)	0.104		
History of malignant tumor	49 (7.47)	27 (5.27)	0.164		
Family history of lung cancer	57 (8.69)	52 (10.16)	0.451		
Hypertension	165 (25.15)	106 (20.70)	0.086		
Diabetes	99 (15.09)	21 (4.10)	< 0.001	0.555 (0.539–0.571)	< 0.001
Tumor markers^&^					
CEA	18 (2.74)	20 (3.91)	0.345		
CYFRA 21-1	97 (14.79)	68 (13.28)	0.517		
NSE	15 (2.29)	19 (3.71)	0.207		
pro-GRP	27 (4.12)	13 (2.54)	0.191		
SCC-Ag	6 (0.91)	7 (1.37)	0.652		

Note: Values are expressed as a number (%) or the mean ± SD. Characteristics with *p* < 0.05 in univariate analysis were further included in ROC analysis

^&^Reference ranges for tumor markers: CEA, 0–5 ng/mL; CYFRA 21-1, 0–2.08 ng/mL; NSE, 0–16.3 ng/mL; pro-GRP, 25.3–77.8 ng/mL; SCC-Ag, 0–1.5 ng/mL

*SBSNs* subcentimeter benign solid nodules, *SMSNs* subcentimeter malignant solid nodules, *ROC* receiver operating characteristic, *AUC* area under the curve, *CI* confidence interval, *CEA* carcinoembryonic antigen, *CYFRA 21-1* cytokeratin 19 antigen, *NSE* neuronal-specific enolase, *pro-GRP* gastrin-releasing peptide precursor, *SCC-Ag* squamous cell carcinoma antigen

Tables 3 and S4 detail the CT features of lesions in the training and validation sets, respectively. In the training set, lesions with heterogeneous density (51.84% vs 7.00%, *p* < 0.001), coarse margin (38.91% vs 28.87%, *p* < 0.001), lobulation (31.97% vs 14.37%, *p* < 0.001), spiculation (11.29% vs 6.88%, *p* = 0.003), and air bronchogram sign (15.24% vs 10.32%, *p* = 0.005) were more frequent in SMSNs, while those with ill-defined boundary (8.35% vs 4.35%, *p* = 0.002), pleural indentation (17.57% vs 11.29%, *p* < 0.001), halo sign (8.72% vs 1.09%, *p* < 0.001), and calcification (1.72% vs 0%, *p* < 0.001) were more common in SBSNs. The ROC analysis and DeLong test showed that heterogeneous density achieved the highest AUC among the CT indicators for differentiation in both the training (0.722, 95% CI: 0.676–0.769; *p* < 0.001) and validation sets (AUC: 0.738, 95% CI: 0.696–0.777; *p* < 0.001). The collinearity between density homogeneity and lobulation or spiculation sign was weak (Cramér’s *V* values: 0.072, *p* = 0.005; 0.099, *p* < 0.001).

**Table 3:** Comparison of the CT features of SBSNs and SMSNs in the training set

Characteristics	SBSNs	SMSNs	*p*-value	ROC analysis	
	(*n* = 814)	(*n* = 735)		AUC (95% CI)	*p*-value
Diameter (range, mm)	7.25 ± 1.58 (4.03–10)	7.29 ± 1.79 (3.91–10)	0.249		
Location			0.564		
Upper lobe	478 (58.72)	420 (57.14)			
Middle or lower lobe	336 (41.28)	315 (42.86)			
Density homogeneity			< 0.001		
Homogeneous	757 (93.00)	354 (48.16)			
Heterogeneous	57 (7.00)	381 (51.84)		0.722 (0.676–0.769)	< 0.001
Shape			0.494		
Round/oval	780 (95.82)	698 (94.97)			
Irregular	34 (4.18)	37 (5.03)			
Boundary			0.002		
Well-defined	746 (91.65)	703 (95.65)			
Ill-defined	68 (8.35)	32 (4.35)			0.051
Margin			< 0.001		
Smooth	579 (71.13)	449 (61.09)			
Coarse	235 (28.87)	286 (38.91)		0.550 (0.527–0.574)	0.003
Lobulation	117 (14.37)	235 (31.97)	< 0.001	0.572 (0.520–0.624)	< 0.001
Spiculation	56 (6.88)	83 (11.29)	0.003	0.531 (0.478–0.584)	0.032
Pleural indentation	143 (17.57)	83 (11.29)	< 0.001	0.549 (0.497–0.602)	< 0.001
Vacuole sign	35 (4.30)	44 (5.99)	0.164		0.134
Air bronchogram sign	84 (10.32)	112 (15.24)	0.005		0.063
Halo sign	71 (8.72)	8 (1.09)	< 0.001	0.543 (0.490–0.595)	< 0.001
Calcification	14 (1.72)	0 (0.00)	< 0.001		0.356

Note: Values are expressed as a number (%) or the mean ± SD. Characteristics with *p* < 0.05 in univariate analysis were further included in ROC analysis

*SBSNs* subcentimeter benign solid nodules, *SMSNs* subcentimeter malignant solid nodules, *ROC* receiver operating characteristic, *AUC* area under the curve, *CI* confidence interval

### The optimal cutoff threshold of the SD of mean CT value for differentiating heterogeneous and homogeneous SNs

In the training set, the mean CT value of heterogeneous SNs (−33.62 ± 67.09 HU) was significantly lower than that of homogeneous ones (1.14 ± 63.53 HU), while the SD of mean CT value of heterogeneous SNs (80.22 ± 27.65 HU) was significantly higher than that of homogeneous ones (35.11 ± 11.20 HU). The optimal cutoff point of the SD of mean CT value for differentiating visually heterogeneous and homogeneous SNs was 57.3 HU, which achieved an AUC of 0.938 (95% CI: 0.923–0.952, *p* < 0.001), with a sensitivity of 0.774 (95% CI: 0.735–0.813) and a specificity of 0.953 (95% CI: 0.941–0.966). In the validation set, the differential performance of the cutoff point was re-evaluated and achieved an AUC of 0.928 (95% CI: 0.896–0.956), sensitivity of 0.890 (95% CI: 0.834–0.949), specificity of 0.960 (95% CI: 0.935–0.980), accuracy of 0.944 (95% CI: 0.921–0.963), positive predictive value (PPV) of 0.888 (95% CI: 0.826–0.941), and negative predictive value (NPV) of 0.963 (95% CI: 0.940–0.983).

### Mixed-effects logistic regression analysis for predicting SMSNs based on clinical and CT features

Mixed-effects logistic regression analysis for predicting SMSNs was performed based on clinical and CT features (excluding density homogeneity) selected by LASSO regression analysis. The model based on the training set (Model A-Training) revealed that lobulation (odds ratio [OR]: 2.857; 95% CI: 2.176–3.752; *p* < 0.001), spiculation (OR: 1.707; 95% CI: 1.115–2.612; *p* = 0.014), and air bronchogram sign (OR: 1.431; 95% CI: 1.029–1.991; *p* = 0.033) were independent predictors of SMSNs. In contrast, diabetes (OR: 4.167; 95% CI: 2.682–6.459; *p* < 0.001), pleural indentation (OR: 2.137; 95% CI: 1.527–2.994; *p* < 0.001) and halo sign (OR: 8.981; 95% CI: 4.182–19.285; *p* < 0.001) were independent predictors of SBSNs.

The optimism-corrected AUC of Model A-Training was 0.698 (95% CI: 0.692–0.704; *p* < 0.001), with an accuracy of 0.640 (95% CI: 0.624–0.651), sensitivity of 0.673 (95% CI: 0.535–0.833), specificity of 0.610 (95% CI: 0.440–0.743), PPV of 0.612 (95% CI: 0.574–0.650), NPV of 0.678 (95% CI: 0.637–0.745), Brier score of 0.217 (95% CI: 0.216–0.218), and calibration slope/intercept of 1.078/−0.023. The model based on the validation set (Model A-Validation) achieved an optimism-corrected AUC of 0.679 (95% CI: 0.656–0.694; *p* < 0.001), with an accuracy of 0.641 (95% CI: 0.587–0.685), sensitivity of 0.608 (95% CI: 0.374–0.847), specificity of 0.663 (95% CI: 0.420–0.881), PPV of 0.562 (95% CI: 0.491–0.694), NPV of 0.723 (95% CI: 0.677–0.803), Brier score of 0.216 (95% CI: 0.212–0.222), and calibration slope/intercept of 1.026/0.005.

**Fig. 3:**
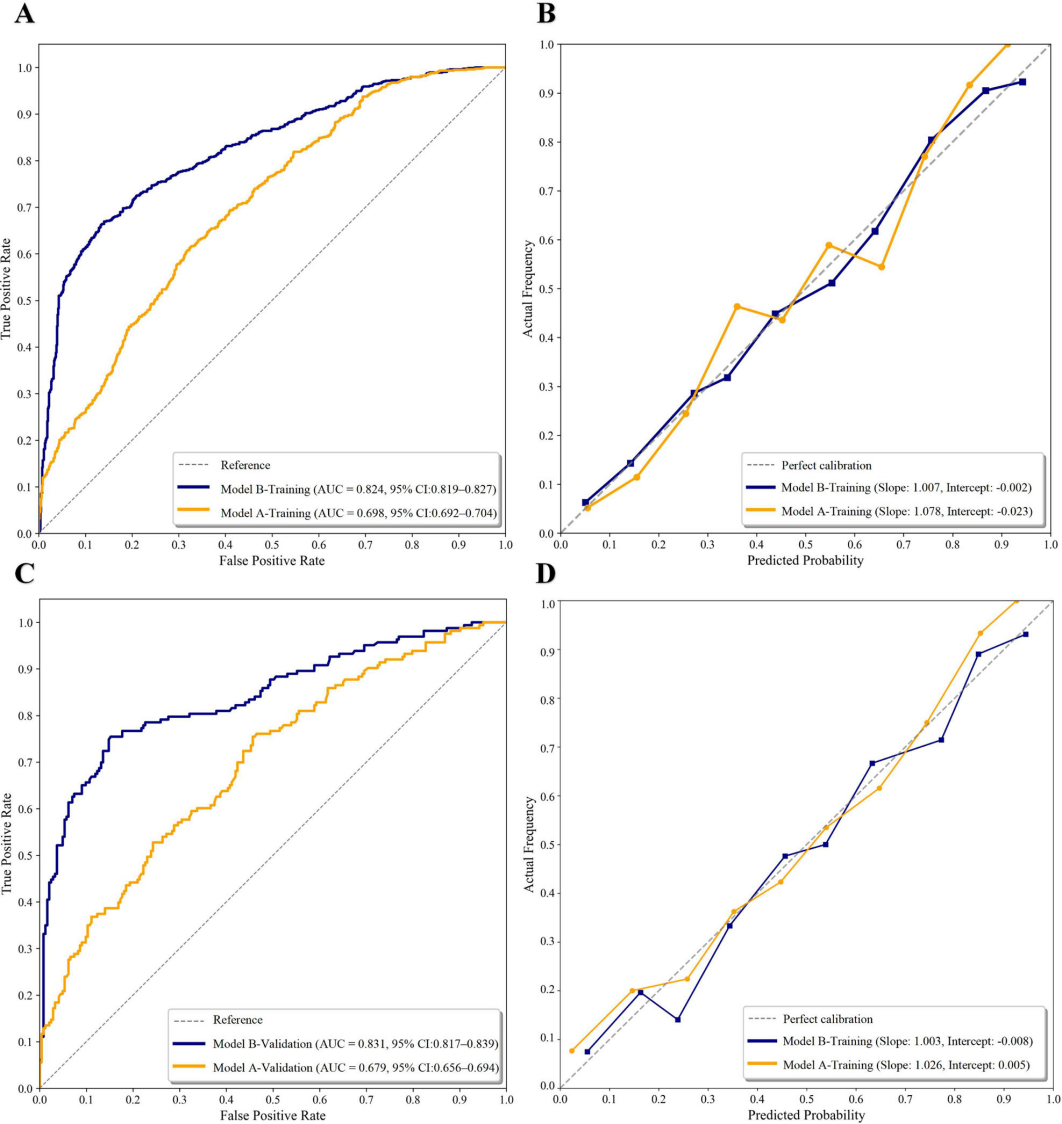
ROC and calibration curves of models in the training (**A**, **B**) and validation set (**C**, **D**). After incorporating the indicator of density homogeneity (Model B), the performance of the predictive model (Model A) significantly improved in both the training (AUC: 0.824 vs 0.698, *p* < 0.001) and validation set (AUC: 0.831 vs 0.679, *p* < 0.001). ROC, receiver operating characteristic; AUC, area under the curve

Model B-Training was developed based on patients’ clinical characteristics and CT features of lesions (including density homogeneity) in the training set. It revealed that heterogeneous density (OR: 15.794; 95% CI: 11.277–22.120; *p* < 0.001), lobulation (OR: 2.818; 95% CI: 2.059–3.866; *p* < 0.001), spiculation (OR: 3.109; 95% CI: 1.873–5.176; *p* < 0.001), and air bronchogram sign (OR: 1.462; 95% CI: 0.942–2.145; *p* = 0.048) were independent predictors of SMSNs. In contrast, ill-defined boundary (OR: 2.740; 95% CI: 1.517–5.097; *p* < 0.001), pleural indentation (OR: 2.151; 95% CI: 1.516–3.276; *p* < 0.001), halo sign (OR: 6.803; 95% CI: 3.285–18.965; *p* < 0.001), and diabetes (OR: 3.305; 95% CI: 2.095–5.708; *p* < 0.001) were independent predictors of SBSNs. It achieved an optimism-corrected AUC of 0.824 (95% CI: 0.819–0.827; *p* < 0.001), with an accuracy of 0.767 (95% CI: 0.759–0.775), sensitivity of 0.665 (95% CI: 0.610–0.730), specificity of 0.859 (95% CI: 0.749–0.905), PPV of 0.812 (95% CI: 0.761–0.855), NPV of 0.740 (95% CI: 0.720–0.765), Brier score of 0.165 (95% CI: 0.164–0.167), calibration slope/intercept of 1.007/−0.002. Compared with Model A-Training, the efficiency of Model B-Training in predicting SMSNs significantly increased (*p* < 0.001) (Fig. 3), with an NRI of 48.61% (95% CI: 43.98–53.68%).

Model B-Validation was developed based on the validation set. It revealed that heterogeneous density had the highest OR (20.296; 95% CI: 11.519–55.105; *p* < 0.001) and achieved an optimism-corrected AUC of 0.831 (95% CI: 0.817–0.839; *p* < 0.001), with an accuracy of 0.806 (95% CI: 0.788–0.820), sensitivity of 0.704 (95% CI: 0.629–0.767), specificity of 0.874 (95% CI: 0.815–0.930), PPV of 0.792 (95% CI: 0.731–0.858), NPV of 0.816 (95% CI: 0.788–0.839), Brier score of 0.152 (95% CI: 0.147–0.159), and calibration slope/intercept of 1.003/−0.008. Compared with Model A-Validation, the efficiency of Model B-Validation also significantly increased (*p* < 0.001), with an NRI of 56.89% (95% CI: 44.45%–69.90%) (Fig. 3).

### Pathological diagnosis of heterogeneous and homogeneous SMSNs

**Fig. 4:**
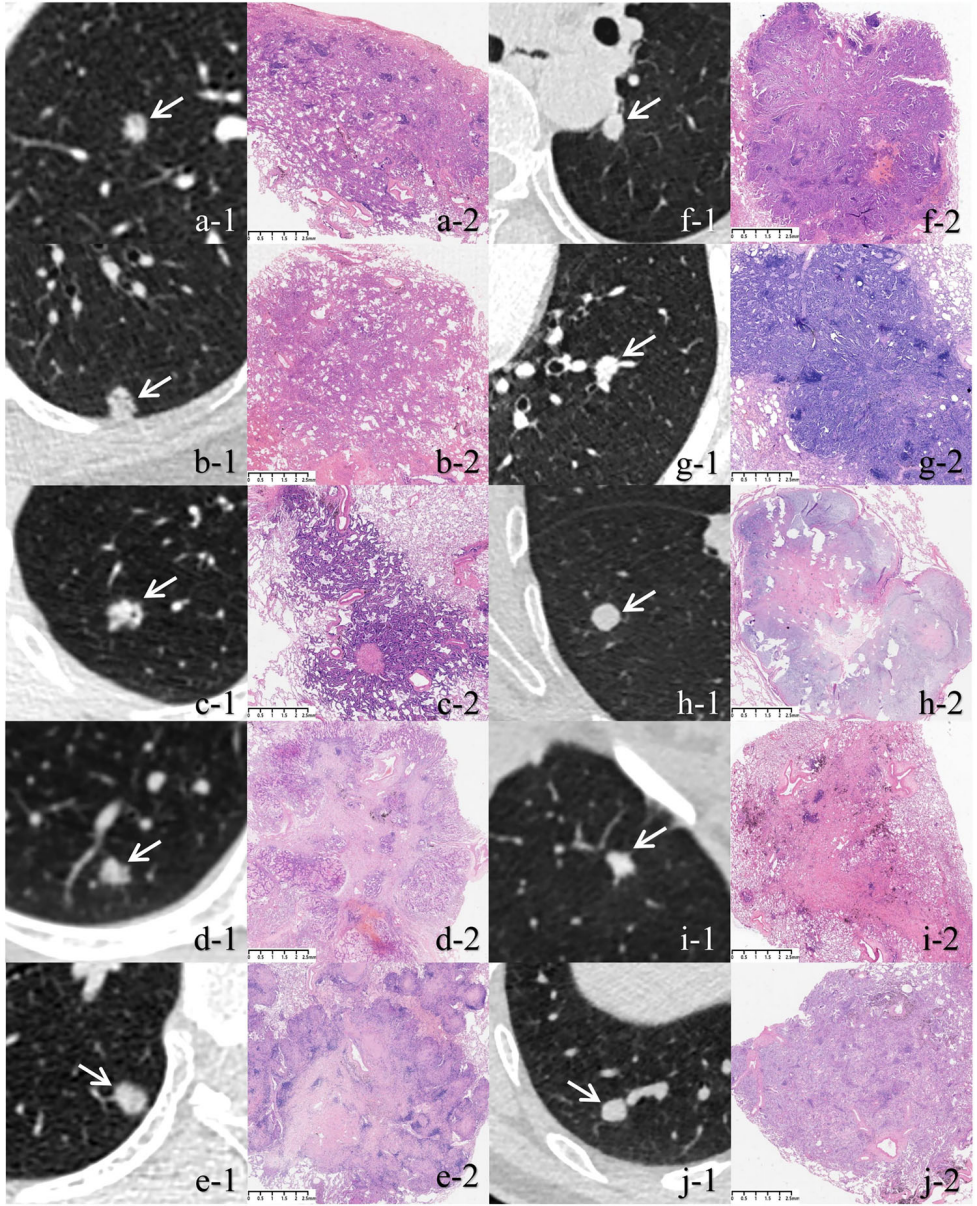
Representative paired CT-pathology images of heterogeneous (**a**–**e**) and homogeneous SNs (**f**–**j**). **a** Axial CT image shows a heterogeneous SN (SD: 102.47 HU) (a-1), pathologically confirmed as an IAC. Photomicrograph (a-2; H&E stain; original magnification, ×10) reveals abundant residual air spaces dispersed throughout invasive growth components. **b** Axial CT image shows a heterogeneous SN (SD: 108.34 HU) (b-1), pathologically confirmed as a MIA. Photomicrograph (b-2; H&E stain; original magnification, ×10) demonstrates interspersed invasive and lepidic growth patterns. **c** Axial CT image shows a heterogeneous SN (SD: 123.52 HU) (c-1), pathologically confirmed as a MIA. Photomicrograph (c-2; H&E stain; original magnification, ×10) shows invasive growth components intermixed with focal fibrous tissue. **d** Axial CT image shows a heterogeneous SN (SD: 99.71 HU) (d-1), pathologically confirmed as a MAC. Photomicrograph (d-2; H&E stain; original magnification, ×10) shows abundant mucin surrounded by invasive growth components. **e** Axial CT image shows a heterogeneous SN (SD: 65.79 HU) (e-1), pathologically confirmed as a granuloma. Photomicrograph (e-2; H&E stain; original magnification, ×10) shows extensive infiltrating inflammatory cells with focal areas of necrosis. **f** Axial CT image shows a homogeneous SN (SD: 41.02 HU) (f-1), pathologically confirmed as an IAC. Photomicrograph (f-2; H&E stain; original magnification, ×10) shows interspersed multiple invasive growth patterns, including acinar, papillary, micropapillary, and cribriform subtypes. **g** Axial CT image shows a homogeneous SN (SD: 51.24 HU) (g-1), pathologically confirmed as a MIA. Photomicrograph (g-2; H&E stain; original magnification, ×10) shows acinar growth patterns accounting for approximately 95% of the entire lesion. **h** Axial CT image shows a homogeneous SN (SD: 34.77 HU) (h-1), pathologically confirmed as a hamartoma. Photomicrograph (h-2; H&E stain; original magnification, ×10) shows compact tumor cell architecture. **i** Axial CT image shows a homogeneous SN (SD: 42.18 HU) (i-1), pathologically confirmed as fibrous-hyaline degeneration. Photomicrograph (i-2; H&E stain; original magnification, ×10) shows dense fibrous tissue with scattered inflammatory cell infiltration. **j** Axial CT image shows a homogeneous SN (SD: 28.95 HU) (j-1), pathologically confirmed as fibrous-hyaline degeneration. Photomicrograph (j-2; H&E stain; original magnification, ×10) shows dense fibrous tissue with scattered inflammatory cell infiltration. CT, computed tomography; SN, solid nodule; SD, standard deviation (of the lesion’s mean CT value); HU, Hounsfield units; IAC, invasive adenocarcinoma; H&E, hematoxylin-eosin; MIA, minimally invasive adenocarcinoma; MAC, mucinous adenocarcinoma

Table 4 summarizes the pathological diagnosis of homogeneous and heterogeneous SMSNs in the training set. Non-mucinous adenocarcinomas (non-MACs) were significantly more common in heterogeneous SMSNs compared with homogeneous ones (95.01% vs 85.59%, *p* < 0.001). Invasive adenocarcinoma (IACs) (65.54% vs 40.68%, *p* < 0.001), MACs (7.91% vs 3.94%, *p* = 0.033), and squamous cell carcinomas (SCCs) (5.93% vs 1.05%, *p* < 0.001) were significantly more common in homogeneous SMSNs, while minimally invasive adenocarcinoma (MIAs) (30.45% vs 9.89%, *p* < 0.001) and adenocarcinomas in situ (AISs) (23.88% vs 10.17%, *p* < 0.001) were more common in heterogeneous ones (Fig. 4). Additionally, among non-MACs, lesions with lepidic growth pattern were significantly more common in those presenting as heterogeneous nodules compared with those as homogeneous nodules (66.40% vs 35.59%, *p* < 0.001).

**Table 4:** Comparison of pathological diagnosis of homogeneous and heterogeneous SMSNs

Pathological diagnosis	Heterogeneous SMSN (*n* = 381)	Homogeneous SMSN (*n* = 354)	*p*-value
Non-MACs	362 (95.01)	303 (85.59)	< 0.001
AISs	91 (23.88)	36 (10.17)	< 0.001
MIAs	116 (30.45)	35 (9.89)	< 0.001
IACs	155 (40.68)	232 (65.54)	< 0.001
MACs	15 (3.94)	28 (7.91)	0.033
SCCs	4 (1.05)	21 (5.93)	< 0.001
SCLCs	0 (0.00)	2 (0.56)	0.447

Note: Values are expressed as a number (%)

*non-MACs* non-mucinous adenocarcinomas, *AISs* adenocarcinomas in situ, *MIAs* minimally invasive adenocarcinomas, *IACs* invasive adenocarcinomas, *MACs* mucinous adenocarcinomas, *SCCs* squamous cell carcinomas, *SCLCs* small cell lung cancers

### Independent predictors of SMSNs in different diameter ranges

**Fig. 5:**
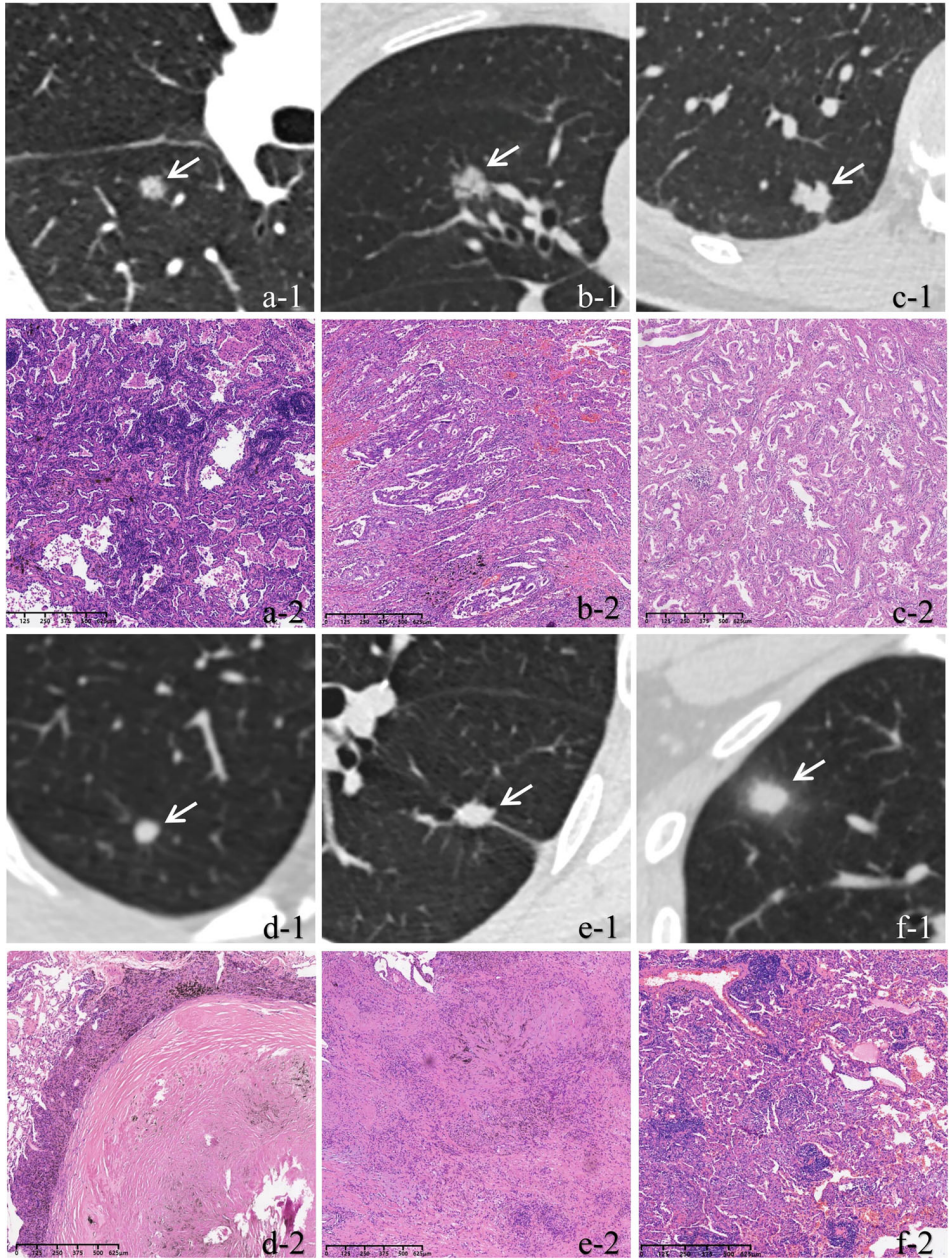
**a** A 42-year-old female with a SMSN. (a-1) Axial CT image demonstrates a 5.8-mm well-defined SN (SD: 64.54 HU) with heterogeneous density in the right lower lobe. Postoperative histopathologic examination confirms MIA. (a-2) Photomicrograph of the specimen shows the lesion consisting of lepidic growth pattern with significant fibrous proliferation (H&E stain; original magnification, ×40). **b** A 54-year-old female with a SMSN. (b-1) Axial CT image demonstrates a 7.9-mm SN (SD: 91.45 HU) with heterogeneous density, lobulation, and air bronchogram sign in the right middle lobe. Postoperative histopathologic examination confirms IAC. (b-2) Photomicrograph of the specimen shows the lesion predominantly consisting of invasive components with minor lepidic elements (H&E stain; original magnification, ×40). **c** A 51-year-old female with a SMSN. (c-1) Axial CT image demonstrates a 9.7-mm well-defined SN (SD: 30.37 HU) with homogeneous density, lobulation, and pleural indentation in the right lower lobe. Postoperative histopathologic examination confirms IAC. (c-2) Photomicrograph of the specimen shows the lesion is mainly composed of invasive components with minimal lepidic components (H&E stain; original magnification, ×40). **d** A 43-year-old female diabetic with a SBSN. (d-1) Axial CT image shows a 4.7-mm well-defined SN (SD: 35.40 HU) with homogeneous density in the right upper lobe. Postoperative histopathologic examination confirms fibrous-hyaline degeneration. (d-2) Photomicrograph of the specimen shows marked fibrous proliferation in the lesion (H&E stain; original magnification, ×40). **e** A 39-year-old male diabetic with a SBSN. (e-1) Axial CT image shows a 7.7-mm SN (SD: 44.29 HU) with homogeneous density, coarse margin, and pleural indentation in the left lower lobe. Postoperative histopathologic examination confirms granuloma. (e-2) Photomicrograph of the specimen shows a granuloma with infiltration of inflammatory cells and fibrosis (H&E stain; original magnification, ×40). **f** A 35-year-old male diabetic with a SBSN. (f-1) Axial CT image shows a 9.4-mm ill-defined SN (SD: 11.46 HU) with homogeneous density and halo sign in the right upper lobe. Postoperative histopathologic examination confirms granuloma. (f-2) Photomicrograph of the specimen shows a granuloma with extensive infiltration of inflammatory cells (H&E stain; original magnification, ×40). SMSN, subcentimeter malignant solid nodule; CT, computed tomography; SD, standard deviation of the lesion’s mean CT value; HU, Hounsfield units; MIA, minimally invasive adenocarcinoma; H&E, hematoxylin and eosin; IAC, invasive adenocarcinoma; SBSN, subcentimeter benign solid nodule

Figure S2 shows the distribution of heterogeneous and homogeneous nodules in SBSNs and SMSNs across different diameter ranges (Group I: ≤ 6 mm; Group II: > 6 mm but ≤ 8 mm; and Group III: > 8 mm). In the three groups, the proportions of heterogeneous nodules in SMSNs (40.68%–60.52%) were significantly higher than those in SBSNs (6.27%–8.52%) (each *p* < 0.001) (Fig. 5).

**Fig. 6:**
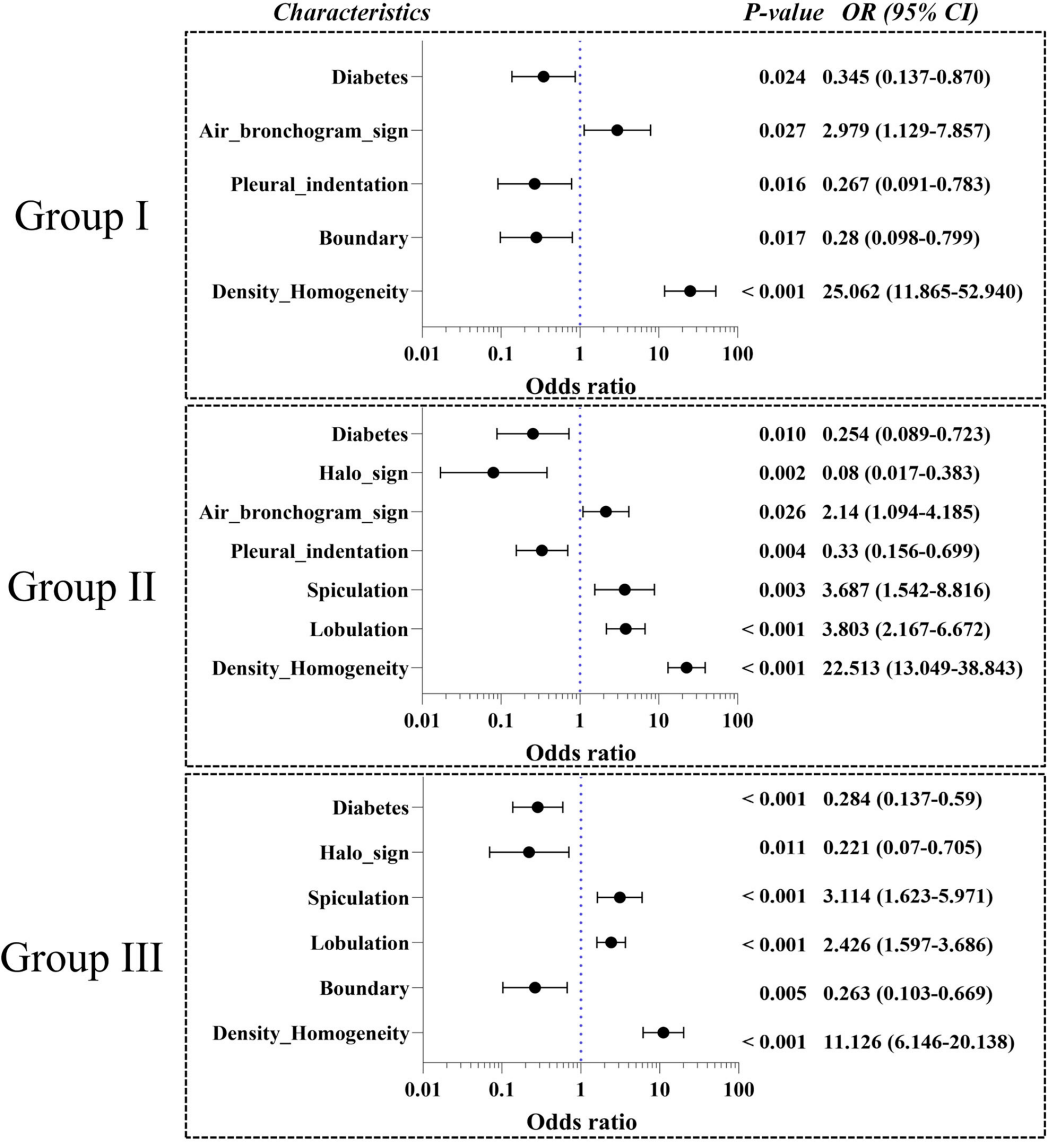
Independent predictors of SMSNs in Groups I–III. The black dots refer to the ORs of characteristics, and the horizontal lines through dots indicate the 95% CI. ORs are expressed as a number (95% CI). ORs, odds ratios; CI, confidence interval

Figure 6 presents the independent clinical and CT predictors of SMSNs identified by logistic regression analysis across Groups I–III. Heterogeneous density was an independent predictor of SMSNs in all groups, demonstrating the highest ORs (11.126–25.062). Both lobulation and spiculation were independent predictors of SMSNs in Groups II and III, and air bronchogram sign was in Groups I and II. In contrast, diabetes was an independent predictor of SBSNs in all three groups; ill-defined boundary was in Groups I and III; pleural indentation was in Groups I and II; and halo sign was in Groups II and III. The AUCs of predictive models in Groups I, II, and III were 0.838 (95% CI: 0.823–0.847; *p* < 0.001), 0.866 (95% CI: 0.856–0.875; *p* < 0.001), and 0.799 (95% CI: 0.789–0.804; *p* < 0.001), respectively.

## Discussion

Clinical characteristics and traditional CT features play a significant role in differentiating solid pulmonary nodules and masses. However, the present study found that their value in differentiating subcentimeter SNs was limited and heterogeneous density is an important indicator. After incorporating density homogeneity, the efficiency of the predictive model was significantly enhanced, and heterogeneous density served as the most important predictor of SMSNs. Furthermore, across three different diameter groups, density homogeneity consistently demonstrated greater differential significance, especially in the smaller subcentimeter lesions. Therefore, density homogeneity should be considered a stable and reliable radiological predictor of malignancy among subcentimeter SNs. The smaller subcentimeter heterogeneous SNs are at a higher risk of malignancy and require close surveillance for early diagnosis and treatment.

Among the clinical and laboratory indicators, diabetes was the only predictor of SBSNs. Diabetes is often associated with a weakened immune system. Therefore, patients with diabetes are at a higher risk of pulmonary infections and inflammatory reactions, which can lead to the formation of granulomas and nonspecific inflammatory nodules [23, 24]. Although smoking history is a well-known high-risk factor for lung cancer [25], it was not a significant indicator of SMSNs in this study, possibly due to the higher proportion of female participants among patients with SMSNs, a population with a lower prevalence of smoking. Furthermore, none of the tumor markers demonstrated predictive value for SMSNs. This may be because the levels of these markers typically increase with larger tumor size [26, 27], and thus, they are not significantly elevated in subcentimeter stages. Consequently, when differentiating subcentimeter SNs, it is important to assess patients’ potential immunosuppressive conditions.

In addition, several traditional CT features were also found to be helpful for differentiating SMSNs and SBSNs. Previous studies have reported that lobulation and spiculation are more obvious in larger lung cancers [12]. In the present study, they were more frequently observed in SMSNs > 6 mm. It is plausible that these two features could possess stronger differential power in larger SNs. Additionally, these two features exhibited no significant collinearity with density homogeneity. Interestingly, although pleural indentation is a recognized indicator of peripheral lung cancers [28], it was more common in SBSNs ≤ 8 mm in this study. This finding may be related to the fact that pleural indentation is primarily caused by contractile effects of fibrous proliferation in lesions [22, 29]. In this study, the SBSNs were predominantly granulomas and fibro-hyaline degenerative lesions, which are characterized by extensive fibrotic proliferation [30, 31]. However, for nodules > 8 mm, no significant difference in this sign was observed between SMSNs and SBSNs, further indicating that fibrous proliferation in smaller malignant nodules is relatively mild but becomes more significant as the tumor grows. Therefore, the malignancy indicative value of pleural indentation should be interpreted cautiously among subcentimeter SNs, especially for those ≤ 8 mm. Additionally, air bronchogram sign, typically associated with well-differentiated adenocarcinomas [32], was more frequently observed in SMSNs ≤ 8 mm. We suppose this could result from the low invasiveness of these small SNs, which is insufficient to generally obscure the airways within lesions. Therefore, while these features may serve as predictors of SMSNs, their discriminative value is limited, highlighting the need for additional indicators to achieve more accurate differentiation of small SNs.

In contrast to clinical characteristics and traditional CT features, density homogeneity demonstrated substantial diagnostic efficiency, which may be associated with the histopathological heterogeneity between SMSNs and SBSNs. In the present study, most SMSNs were non-MACs. Among these lesions, heterogeneous SMSNs were more likely to be MIAs or AISs, while most homogeneous ones were IACs. Moreover, heterogeneous SMSNs were more likely to contain lepidic growth pattern compared with homogeneous ones. Thus, it is possible that the density of heterogeneous SMSN may progressively increase with the advancement of invasive pathological components progressing and fibrous tissue proliferation, ultimately resulting in a radiologically homogeneous appearance. In addition, the mixed distribution of invasive components, mucus, fibrous tissue, or residual airspace may also be associated with heterogeneous density on CT images. In contrast, granulomas or fibrous tissue can form within a month and may present as chronic lesions demonstrating dense and homogeneous architecture [33]. The combination of these factors leads to homogeneous density in SBSNs on CT imaging.

A previous study has identified density homogeneity as one of the predictors for differentiating SNs [10]. However, to the best of our knowledge, the discriminative significance of density homogeneity in subcentimeter SNs, superior to that of other morphological CT features, has not been fully revealed. Furthermore, previous studies have reported that nodules with higher skewness and kurtosis (histogram parameters) are associated with an increased malignant risk and greater invasiveness [34, 35]. To a certain extent, the present results are consistent with these findings, as increased skewness and kurtosis are associated with more significant density heterogeneity. Based on the present data, we propose that density homogeneity should be regarded as a primary factor for the differential diagnosis of subcentimeter SNs. Following visual assessment, the optimal cutoff threshold for the SD of mean CT value in determining the homogeneity of SNs was 57.3 HU, which further demonstrated excellent discriminatory performance in the validation set. Compared with subjective visual assessment, this quantitative parameter is more objective. Therefore, we recommend that the SD of mean CT value serves as an important criterion for evaluating and verifying the homogeneity of SNs, especially for those indeterminate ones at visual assessment.

Among all three diameter groups, density homogeneity exhibited the highest discriminatory efficiency, particularly in smaller subcentimeter SNs. Notably, the proportion of heterogeneous nodules decreased in SMSNs > 8 mm, further suggesting that the density of SMSNs tends to progressively increase during natural progression, ultimately manifesting as radiologically homogeneous lesions. Consequently, assessing dynamic changes in lesion density during follow-up may also be helpful for identifying malignant SNs. A transition from heterogeneous to homogeneous density in subcentimeter SNs may indicate an increased risk of malignancy.

This study has several limitations that should be acknowledged. First, the retrospective design may introduce selection bias, and the prospective validation in the future is warranted. Second, density homogeneity in SNs is a subjective radiological indicator based on the lung window, and it was not assessed using the mediastinal window in this study. Therefore, some heterogeneous SNs may be classified as subsolid nodules. In addition to the visual criteria, we introduced a quantitative CT threshold (a SD of mean CT value above 57.3 HU) to define heterogeneous SNs. This cutoff point achieved an AUC of 0.928 in differentiating homogenous and heterogeneous SNs in the validation set. Third, as this study solely assessed SNs ≤ 10 mm, the diagnostic significance of density homogeneity for larger lesions remains undetermined. Fourth, the minor variations in scanning parameters among multiple CT scanners may potentially affect the evaluation of density homogeneity. Fifth, although CT values were measured on the most representative axial slice of each lesion, such planar measurements may not fully capture the overall heterogeneity of the entire nodule. Finally, we did not conduct a follow-up study on the dynamic changes of heterogeneous SNs, which could further reveal the progression patterns of subcentimeter heterogeneous SNs.

## Conclusion

To distinguish subcentimeter SNs, density homogeneity should be fully considered. Heterogeneous subcentimeter SNs, especially those with lobulation, spiculation, or air bronchogram sign, should be highly suspected of malignancy and warrant more aggressive management strategies, such as shorter-interval follow-up or tissue biopsy. For SNs, the SD of mean CT value > 57.3 HU may be an objective reference for identifying heterogeneous ones, although further validation is warranted in future studies.

## Supplementary information

ELECTRONIC SUPPLEMENTARY MATERIAL

## Data Availability

The datasets generated during the current study are not publicly available due to our institutional regulation, but are available from the corresponding author on reasonable request.

## Abbreviations

AUC: Area under the curve
CEA: Carcinoembryonic antigen
CI: Confidence interval
CT: Computed tomography
CYFRA: Cytokeratin 19 antigen 21-1
HU: Hounsfield Unit
LASSO: Least absolute shrinkage selection operator
MACs: Mucinous adenocarcinomas
MIAs: Minimally invasive adenocarcinomas
non-MACs: Non-mucinous adenocarcinomas
NPV: Negative predictive value
NRI: Net reclassification index
NSE: Neuronal-specific enolase
OR: Odds ratio
PACS: Picture archiving and communication system
PPV: Positive predictive value
pro-GRP: Gastrin-releasing peptide precursor
ROC: Receiver operating characteristic
ROI: Region of interest
SBSNs: Subcentimeter benign SNs
SCC-Ag: Squamous cell carcinoma antigen
SCCs: Squamous cell carcinomas
SCLCs: Small cell lung cancer
SD: Standard deviation
SMSNs: Subcentimeter malignant SNs
SNs: Solid nodules
TSCT: Thin-section CT

## References

[1] Bray F, Laversanne M, Sung H et al (2024) Global cancer statistics 2022: GLOBOCAN estimates of incidence and mortality worldwide for 36 cancers in 185 countries. CA Cancer J Clin 74:229–263 38572751 10.3322/caac.21834

[2] Callister ME, Baldwin DR (2016) How should pulmonary nodules be optimally investigated and managed? Lung Cancer 91:48–55 26711934 10.1016/j.lungcan.2015.10.018

[3] Berry MF, Gao R, Kunder CA et al (2018) Presence of even a small ground-glass component in lung adenocarcinoma predicts better survival. Clin Lung Cancer 19:e47–e51 28743420 10.1016/j.cllc.2017.06.020

[4] Sun K, You A, Wang B et al (2021) Clinical T1aN0M0 lung cancer: differences in clinicopathological patterns and oncological outcomes based on the findings on high-resolution computed tomography. Eur Radiol 31:7353–7362 33860370 10.1007/s00330-021-07865-2

[5] Li Z, Pan C, Xu W et al (2024) Distinct impacts of radiological appearance on lymph node metastasis and prognosis based on solid size in clinical T1 non-small cell lung cancer. Respir Res 25:96 38383329 10.1186/s12931-024-02727-z PMC10880259

[6] Choi S, Yoon DW, Shin S et al (2024) Importance of lymph node evaluation in ≤2-cm pure-solid non-small cell lung cancer. Ann Thorac Surg 117:586–593 36608755 10.1016/j.athoracsur.2022.11.040

[7] Hattori A, Matsunaga T, Fukui M et al (2024) Oncological characteristics of epidermal growth factor receptor–mutated clinical stage IA lung adenocarcinoma with radiologically pure-solid appearance. J Thorac Cardiovasc Surg 168:685–96.e2 37995863 10.1016/j.jtcvs.2023.11.025

[8] Swensen SJ, Silverstein MD, Ilstrup DM, Schleck CD, Edell ES (1997) The probability of malignancy in solitary pulmonary nodules. Application to small radiologically indeterminate nodules. Arch Intern Med 157:849–855 9129544

[9] Henschke CI, Yankelevitz DF, Mirtcheva R et al (2002) CT screening for lung cancer: frequency and significance of part-solid and nonsolid nodules. AJR Am J Roentgenol 178:1053–1057 11959700 10.2214/ajr.178.5.1781053

[10] Chu Z-g, Zhang Y, Li W-j et al (2019) Primary solid lung cancerous nodules with different sizes: computed tomography features and their variations. BMC Cancer 19:1060 31699047 10.1186/s12885-019-6274-0 PMC6836448

[11] Cui S-L, Qi L-L, Liu J-N et al (2024) A prediction model based on computed tomography characteristics for identifying malignant from benign sub-centimeter solid pulmonary nodules. J Thorac Dis 16:4238–4249 39144338 10.21037/jtd-23-1943 PMC11320228

[12] He XQ, Huang XT, Luo TY, Liu X, Li Q (2024) The differential computed tomography features between small benign and malignant solid solitary pulmonary nodules with different sizes. Quant Imaging Med Surg 14:1348–1358 38415140 10.21037/qims-23-995 PMC10895103

[13] Chen X, Feng B, Chen Y et al (2020) A CT-based radiomics nomogram for prediction of lung adenocarcinomas and granulomatous lesions in patient with solitary sub-centimeter solid nodules. Cancer Imaging 20:45 32641166 10.1186/s40644-020-00320-3 PMC7346427

[14] Chen C, Geng Q, Song G et al (2023) A comprehensive nomogram combining CT-based radiomics with clinical features for differentiation of benign and malignant lung subcentimeter solid nodules. Front Oncol 13:1066360 37007065 10.3389/fonc.2023.1066360 PMC10064794

[15] Winer-Muram HT (2006) The solitary pulmonary nodule. Radiology 239:34–49 16567482 10.1148/radiol.2391050343

[16] Lee HY, Lee KS, Han J et al (2009) Mucinous versus nonmucinous solitary pulmonary nodular bronchioloalveolar carcinoma: CT and FDG PET findings and pathologic comparisons. Lung Cancer 65:170–175 19111932 10.1016/j.lungcan.2008.11.009

[17] Gao F, Li M, Zhang Z et al (2019) Morphological classification of pre-invasive lesions and early-stage lung adenocarcinoma based on CT images. Eur Radiol 29:5423–5430 30903336 10.1007/s00330-019-06149-0

[18] Suzuki K, Kusumoto M, Watanabe S, Tsuchiya R, Asamura H (2006) Radiologic classification of small adenocarcinoma of the lung: radiologic-pathologic correlation and its prognostic impact. Ann Thorac Surg 81:413–419 16427823 10.1016/j.athoracsur.2005.07.058

[19] Gaeta M, Vinci S, Minutoli F et al (2002) CT and MRI findings of mucin-containing tumors and pseudotumors of the thorax: pictorial review. Eur Radiol 12:181–189 11868096 10.1007/s003300100934

[20] Hu B, Ren W, Feng Z et al (2022) Correlation between CT imaging characteristics and pathological diagnosis for subcentimeter pulmonary nodules. Thorac Cancer 13:1067–1075 35212152 10.1111/1759-7714.14363 PMC8977167

[21] Hu AM, Zhao D, Zheng H et al (2016) Preoperative diagnosis in 46 cases of pulmonary sclerosing hemangioma. Chin Med J (Engl) 129:1377–1378 27231179 10.4103/0366-6999.182839 PMC4894052

[22] Bankier AA, MacMahon H, Colby T et al (2024) Fleischner Society: glossary of terms for thoracic imaging. Radiology 310:e232558 38411514 10.1148/radiol.232558 PMC10902601

[23] Lao M, Li C, Li J et al (2019) Opportunistic invasive fungal disease in patients with type 2 diabetes mellitus from Southern China: clinical features and associated factors. J Diabetes Investig 11:731–744 31758642 10.1111/jdi.13183 PMC7232281

[24] Xu HB, Ding C, Zhao M, Lv FJ, Chu ZG (2025) Exploring the key clinical and CT characteristics of granulomas mimicking peripheral lung cancers: a case-control study. Insights Imaging 16:157 40684047 10.1186/s13244-025-02043-0 PMC12276186

[25] Tindle HA, Stevenson Duncan M, Greevy RA et al (2018) Lifetime smoking history and risk of lung cancer: results from the framingham heart study. J Natl Cancer Inst 110:1201–117 29788259 10.1093/jnci/djy041 PMC6235683

[26] Takamochi K, Yoshida J, Nishimura M et al (2004) Prognosis and histologic features of small pulmonary adenocarcinoma based on serum carcinoembryonic antigen level and computed tomographic findings. Eur J Cardiothorac Surg 25:877–883 15082298 10.1016/j.ejcts.2004.01.049

[27] Icard P, Regnard JF, Essomba A et al (1994) Preoperative carcinoembryonic antigen level as a prognostic indicator in resected primary lung cancer. Ann Thorac Surg 58:811–814 7944708 10.1016/0003-4975(94)90755-2

[28] Seki N, Fujita Y, Shibakuki R et al (2007) Easier understanding of pleural indentation on computed tomography. Intern Med 46:2029–2030 18084129 10.2169/internalmedicine.46.0560

[29] Kim HJ, Cho JY, Lee YJ et al (2019) Clinical significance of pleural attachment and indentation of subsolid nodule lung cancer. Cancer Res Treat 51:1540–1548 30913858 10.4143/crt.2019.057 PMC6790827

[30] Bonham CA, Strek ME, Patterson KC (2016) From granuloma to fibrosis: sarcoidosis associated pulmonary fibrosis. Curr Opin Pulm Med 22:484–491 27379967 10.1097/MCP.0000000000000301 PMC5532138

[31] Travis WD, Brambilla E, Rami-Porta R et al (2008) Visceral pleural invasion: pathologic criteria and use of elastic stains: proposal for the 7th edition of the TNM classification for lung cancer. J Thorac Oncol 3:1384–1390 19057261 10.1097/JTO.0b013e31818e0d9f

[32] Wang Z, Zhu W, Yang M et al (2023) Air bronchogram on chest CT in radiological pure-solid appearance lung cancer: Correlation analysis with genetic pathological features and survival outcomes. Eur J Radiol 169:111194 37976762 10.1016/j.ejrad.2023.111194

[33] Ndlovu H, Marakalala MJ (2016) Granulomas and inflammation: host-directed therapies for tuberculosis. Front Immunol 7:434 27822210 10.3389/fimmu.2016.00434 PMC5075764

[34] Kamiya A, Murayama S, Kamiya H et al (2014) Kurtosis and skewness assessments of solid lung nodule density histograms: differentiating malignant from benign nodules on CT. Jpn J Radiol 32:14–21 24248771 10.1007/s11604-013-0264-y

[35] Margerie-Mellon C, Gill RR, Salazar P et al (2020) Assessing invasiveness of subsolid lung adenocarcinomas with combined attenuation and geometric feature models. Sci Rep 10:14585 32883973 10.1038/s41598-020-70316-3 PMC7471897

