# Supplementary material for: Density homogeneity as a crucial CT indicator for differentiating malignant and benign subcentimeter solid pulmonary nodules: A retrospective multi-center study
Source: Insights Imaging. 2026 May 16;17:133. doi: 10.1186/s13244-026-02301-9 (PMC13179974; doi:10.1186/s13244-026-02301-9)
Supplement: Supplementary file 1 — ELECTRONIC SUPPLEMENTARY MATERIAL [file 13244_2026_2301_MOESM1_ESM.pdf]

Density Homogeneity as a Crucial CT Indicator for Differentiating Malignant and Benign Subcentimeter Solid Pulmonary Nodules: A Retrospective Multi-Center Study

ELECTRONIC SUPPLEMENTARY MATERIAL

Table S1: Scanning parameters and acquisition protocols across different CT scanners.

**CT Scanner**	**Reconstruction kernels**	**Tube Voltage (kVp)**	**Tube Current ref (mA)#**	**Slice Thickness (mm)**	**Pitch**
SOMATOM Perspective (Siemens Healthineers)	B40	120	140	1.0	1.0
Discovery CT750 HD (GE Healthcare)	Lung	110	100	0.625	0.975
SOMATOM Definition Flash (Siemens Healthineers)	I50	120	100	0.625	1.2
SOMATOM Force (Siemens Healthineers)	Br54	110	140	1.0	1.2
OPTIMA CT660 (GE Healthcare)	Lung	120	140	0.625	1.0
Aquilion ONE pureViSION (Canon Medical System)	Lung Std Axial	120	50	1.0	1.0

Note: #Tube current is automatically adjusted.

Table S2 Inter- and intra-observer agreement of CT features

Characteristics	Metric (95% CI)
Density homogeneity	
Inter-observer agreement of all radiologists	0.851 (0.827, 0.873)
Intra-observer agreement of Radiologist A	0.926 (0.905, 0.946)
Intra-observer agreement of Radiologist B	0.934 (0.915, 0.953)
Intra-observer agreement of Radiologist C	0.951 (0.934, 0.968)
Diameter	0.924 (0.810, 0.960)
Mean CT value	0.892 (0.823, 0.944)
SD CT value	0.953 (0.922, 0.984)
Shape	0.891 (0.837, 0.944)
Boundary	0.913 (0.869, 0.957)
Margin	0.801 (0.769, 0.832)
Lobulation sign	0.770 (0.734, 0.807)
Spiculation sign	0.901 (0.862, 0.940)
Pleural indentation sign	0.884 (0.848, 0.919)

Vacuole sign	0.973 (0.949, 0.997)
Air bronchogram sign	0.936 (0.909, 0.962)
Halo sign	0.953 (0.912, 0.994)
Calcification	1.000 (1.000, 1.000)

Note: For density homogeneity, both inter- and intra-observer agreement were assessed, and for the other CT features, only inter-observer agreement was evaluated. Metrics represent ICC for continuous variables and kappa for categorical variables. Values are expressed as a number (95 CI%).

Abbreviations: ICC, intraclass correlation coefficient; CI, confidence interval; CT, computed tomography; SD, standard deviation.

Table S3: Comparison of clinical characteristics between patients with SBSNs and SMSNs in the validation set

Characteristics	SBSNs (n = 190)	SMSNs (n = 141)	P-value	ROC analysis AUC (95% CI)	P-value
Age (years)	56.31 ± 10.69	55.01 ± 1263	0.092		
Gender					
Male	104 (54.74)	84 (59.57)	0.380		
Female	86 (45.26)	57 (40.43)			
Smoking history	51 (26.84)	33 (23.40)	0.655		
History of malignant tumor	17 (8.95)	13 (9.22)	1.000		
Family history of lung cancer	23 (12.11)	15 (10.64)	0.811		
Hypertension	41 (21.58)	23 (16.31)	0.290		
Diabetes	26 (13.68)	8 (5.67)	0.028	0.540 (0.507-0.571)	0.096
Tumor markers&					
CEA	5 (2.63)	3 (2.13)	0.768		
CYFRA 21-1	20 (10.53)	17 (12.06)	0.794		
NSE	7 (3.68)	2 (1.42)	0.362		
pro-GRP	2 (1.05)	3 (2.13)	0.736		
SCC-Ag	2 (1.05)	1 (0.71)	1.000		

Note: Values are expressed as a number (%) or the mean ± standard deviation. Characteristics with P < 0.05 in univariate analysis were further included in ROC analysis.

&Reference ranges for tumor markers: CEA, 0–5 ng/mL; CYFRA 21-1, 0–2.08 ng/mL; NSE, 0–16.3 ng/mL; pro-GRP, 25.3–77.8 ng/mL; SCC-Ag, 0–1.5 ng/mL.

Abbreviations: SBSNs, subcentimeter benign solid nodules; SMSNs, subcentimeter malignant solid nodules; ROC, receiver operating characteristic; AUC, area under the curve; CI, confidence interval; CEA, carcinoembryonic antigen; CYFRA 21-1, cytokeratin 19 antigen; NSE, neuronal-specific enolase; pro-GRP, gastrin-releasing peptide precursor; SCC-Ag, squamous cell carcinoma antigen.

Table S4: Comparison of the CT features of SBSNs and SMSNs in the validation set

Characteristics	SBSNs (n = 244)	SMSNs (n = 163)	P-value	ROC analysis AUC (95% CI)	ROC analysis P-value
Diameter (range, mm)	7.65 ± 1.55 (4.23–10)	7.9 ± 1.48 (4.05–10)	0.056		
Location					
Upper lobe	106 (43.44)	85 (52.15)	0.105		
Middle or lower lobe	138 (56.56)	78 (47.85)			
Density homogeneity				0.738 (0.696–0.777)	< 0.001
Homogeneous	227 (93.03)	74 (45.40)	< 0.001		
Heterogeneous	17 (6.97)	89 (54.60)			
Shape					
Round/oval	227 (93.03)	154 (94.48)	0.706		
Irregular	17 (6.97)	9 (5.52)			
Boundary					
Well-defined	234 (95.90)	155 (95.09)	0.886		
Ill-defined	10 (4.10)	8 (4.91)			
Margin				0.547 (0.503–0.591)	0.933
Smooth	56 (22.95)	53 (32.52)	0.043		
Coarse	188 (77.05)	110 (67.48)			
Lobulation	44 (18.03)	47 (28.83)	0.015	0.556 (0.514–0.596)	0.040
Spiculation	10 (4.10)	26 (15.95)	< 0.001	0.559 (0.529–0.589)	< 0.001
Pleural indentation	44 (18.03)	18 (11.04)	0.075		
Vacuole sign	8 (3.28)	6 (3.68)	0.827		
Air bronchogram sign	30 (12.30)	43 (26.38)	< 0.001	0.570 (0.530–0.610)	0.009
Halo sign	18 (7.38)	4 (2.45)	0.054		
Calcification	8 (3.28)	0 (0.00)	0.049	0.516 (0.506–0.528)	0.957

Note: Values are expressed as a number (%) or the mean ± standard deviation.
Characteristics with P < 0.05 in univariate analysis were further included in ROC analysis.
Abbreviations: SBSNs, subcentimeter benign solid nodules; SMSNs, subcentimeter malignant solid nodules; ROC, receiver operating characteristic; AUC, area under the curve; CI, confidence interval.

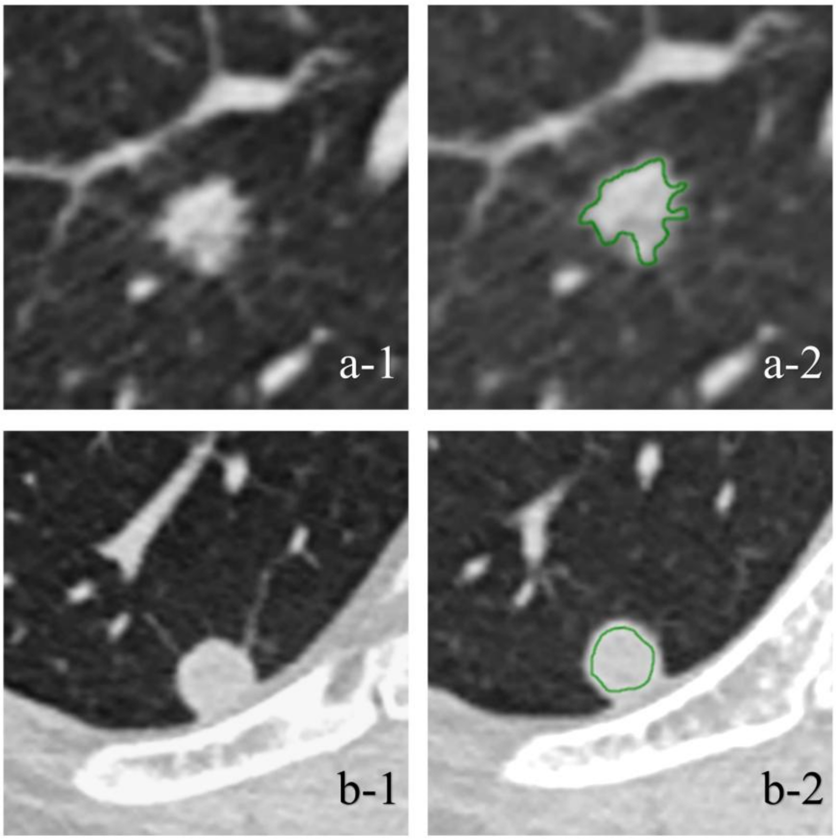

Figure S1: (a-1) A heterogenous SN; (a-2) The corresponding ROI covering approximately 70% of the lesion's area; (b-1) A homogenous SN; (b-2) The corresponding ROI covering approximately 70% of the lesion's area. Abbreviations: SNs, solid nodules; ROI, region of interest.

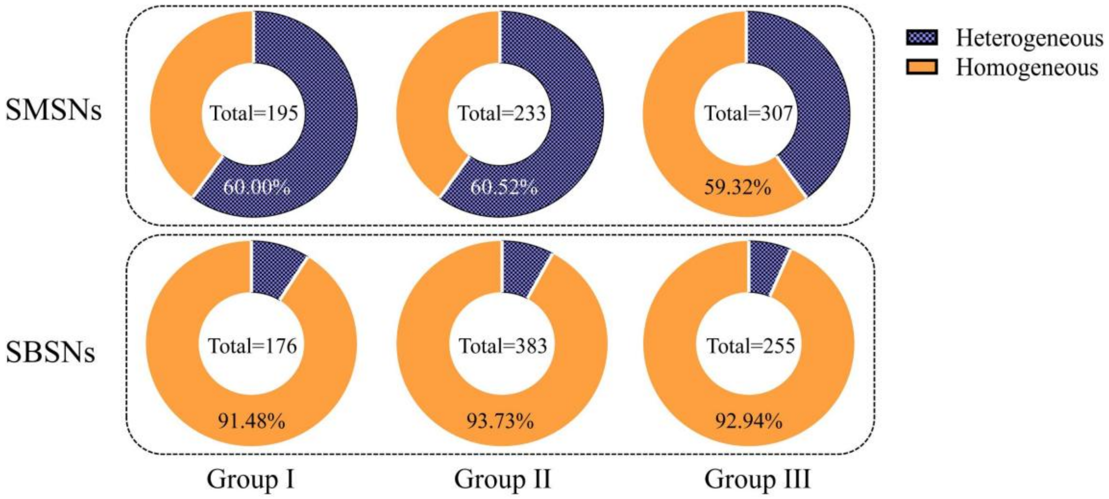

Figure S2: Proportions of heterogeneous and homogeneous lesions in SBSNs and SMSNs in different groups. Abbreviations: SMSNs, subcentimeter malignant solid nodules; SBSNs, subcentimeter benign solid nodules.
